# Case Report: A case of toxic epidermal necrolysis induced by serplulimab: integrated Chinese and Western nursing care

**DOI:** 10.3389/fmed.2026.1759311

**Published:** 2026-02-16

**Authors:** Dan Wang

**Affiliations:** The Third Hospital of Mianyang, Sichuan Mental Health Center, Mianying, Sichuan, China

**Keywords:** cancer, combined Chinese and Western medicine care, nursing experience, skin lesion care, toxic epidermal necrolysis

## Abstract

This study summarizes the integrated Chinese and Western medical nursing experience for a lung cancer patient admitted to our hospital in 2025 who developed toxic epidermal necrolysis (TEN) following treatment with serplulimab. Medical staff provided personalized care addressing the patient’s skin and mucosal lesions, pain, and psychological wellbeing, aiming to establish evidence for the future treatment and management of such symptoms. Following multidisciplinary consultation and 39 days of meticulous treatment and nursing care, the patient’s widespread skin erosion largely healed, with no new rashes observed, ultimately leading to discharge in an improved condition.

## 1 Introduction

With the acceleration of population aging and changes in lifestyle, patterns of cancer incidence and mortality have shifted. As a result, the overall cancer disease burden continues to increase, and the challenges of prevention and control are becoming increasingly severe (1). Globally, lung cancer is undoubtedly the leading malignant tumor in terms of both incidence and mortality (1). Pathologically, small-cell lung cancer (SCLC) accounts for approximately 15% of all lung cancers. It is the most aggressive subtype, characterized by rapid progression, high metastatic potential, and poor prognosis (2). Clinically, SCLC is typically categorized into two main stages: limited stage and extensive stage. The majority of lung cancer patients are already in the extensive stage at diagnosis, resulting in a generally poor overall prognosis (2).

Serplulimab is an innovative antitumor drug and China’s domestically developed PD-1 monoclonal antibody. It tightly binds to PD-1, thereby blocking the interaction between PD-1 and its ligand PD-L1. This action disrupts the immune escape mechanism of tumor cells, thereby enhancing the killing effect of tumor antigen-specific T cells on tumor cells. Serplulimab has exhibited remarkable efficacy and safety in multiple clinical trials (3). As an innovative PD-1 inhibitor, serplulimab was evaluated in the landmark ASTRUM-005 study exploring efficacy and safety in extensive-stage small-cell lung cancer (ES-SCLC). This trial provided high-level evidence-based medical data that reshaped the landscape of first-line ES-SCLC treatment (4). In January 2023, the National Medical Products Administration (NMPA) approved the indication of serplulimab in combination with carboplatin and etoposide for the first-line treatment of ES-SCLC (5).

Due to the similarity of antigens between tumor cells and normal cells, the immune system may attack normal tissues while eliminating tumors, leading to various toxic reactions, including skin toxicity, hematologic toxicity, gastrointestinal toxicity, neurological toxicity, respiratory toxicity, and nephrotoxicity (6). These toxic reactions often manifest as skin-related disorders and colitis, with less common occurrences including hepatitis, pneumonia, myocarditis, endocrine system disorders, neurological disorders, and rheumatologic/immune system disorders. Previous studies and reports have indicated that approximately 9.5% of patients treated with serplulimab experience grade 3 or higher immune-related adverse events (irAEs), with skin-related irAEs being the most common type of irAEs (7). A meta-analysis evaluating the safety of inhibitors such as serplulimab has also indicated that the overall incidence of adverse events ranged between 54 and 76% (8). Skin irAEs were the most frequently observed, occurring in more than 30% of patients and often being the first adverse reaction detected.

Toxic epidermal necrolysis (TEN) is a rare, acute, severe dermatological condition characterized by epidermal necrosis exceeding 30% of the total body surface area (TBSA), with an annual incidence reported at only 1–7 cases per million (9). Its clinical manifestations primarily include blistering, epidermal detachment, and mucositis affecting multiple sites. Drug-induced adverse reactions are common triggers, including anticonvulsants, antidepressants, non-steroidal anti-inflammatory drugs (NSAIDs), anti-infective agents, and the increasingly prevalent targeted therapies (10). TEN represents a critical illness characterized by an abrupt onset and rapid progression. The majority of cases involve extensive epidermal necrosis and desquamation, frequently complicated by systemic organ failure, electrolyte imbalances, and sepsis, resulting in high mortality rates (11–13). Mortality is positively correlated with the extent of skin involvement, making management of cutaneous lesions a major challenge in care.

Traditional Chinese medicine does not classify severe drug eruptions as a distinct disease entity but rather categorizes them under “drug toxicity.” This condition primarily arises from a patient’s constitutional weakness and subsequent invasion by drug toxins. TCM treatment completely leverages the holistic concept, tailoring therapies to each patient’s unique pattern differentiation, thereby enhancing efficacy (14). Existing research has indicated that TCM plays a significant role in the clinical prevention and treatment of skin adverse reactions associated with anticancer drugs (15). Li (16) treated 64 patients with skin adverse reactions using TCM. The results showed that the combined treatment group exhibited superior outcomes in both reduction in rash grading and clinical symptom improvement compared to the control group. Zeng (17) reported favorable clinical efficacy in the treatment of rashes caused by lung cancer-targeted therapies using TCM, with treatment principles focused on clearing heat, drying dampness, detoxifying, and relieving itching.

Both traditional Chinese medicine nursing and Western nursing each possess distinct advantages. Western nursing, guided by modern medical diagnosis and treatment, exhibits certain strengths in controlling inflammation and mitigating toxic reactions. Traditional Chinese medicine nursing, grounded in fundamental TCM theories and incorporating holistic concepts, uses unique dialectical principles and nursing techniques to provide comprehensive care interventions for patients (18). Integrating the strengths of both approaches enables more holistic care for patients experiencing skin-related adverse reactions while simultaneously reducing mortality rates (19). Previous studies have indicated that Xu (19) achieved favorable skin wound healing in patients with skin infections through integrated Chinese and Western nursing interventions. Wei (20) used distinctive TCM nursing care for patients with toxic epidermal necrolysis, ultimately facilitating the recovery of their difficult-to-heal wounds. The state encourages mutual learning between traditional Chinese medicine and Western medicine to leverage their respective strengths and promote the integration of both medical systems. The report of the 19th National Congress of the Communist Party of China also emphasized upholding equal emphasis on traditional Chinese and Western medicine (18). Consequently, integrated Chinese–Western nursing—a distinctive multidisciplinary team-based approach—has gradually gained traction in clinical practice. Demonstrating significant therapeutic efficacy, it has earned recognition and endorsement from both the state and healthcare professionals, making it a key area warranting attention.

In 2025, a patient with severe drug-induced rash and epidermal necrosis covering more than 60% of the body surface area was admitted to our hospital’s oncology department. The treatment and nursing care presented significant challenges. Following a multidisciplinary consultation, the patient was discharged after 6 weeks with all skin lesions fully healed, leaving only residual hyperpigmentation. The experience of integrated Chinese and Western nursing care is reported below.

## 2 Clinical information

### 2.1 Brief medical history

We present the case of a 63-year-old man who was admitted to the hospital with “small-cell lung cancer diagnosed for 2+ months and generalized rash for 1 month” and was diagnosed with “small-cell lung cancer cT4N2Mx extensive stage” after undergoing lung puncture and chest and abdominal enhancement computed tomography (CT). In the 1st and 2nd months after diagnosis, the patient received two cycles of chemotherapy and immunotherapy. The specific regimen consisted of etoposide 100 mg IV on days 1–3, carboplatin 300 mg IV on day 1, and serplulimab 200 mg IV on day 1, administered once every 3 weeks. After two cycles of treatment, head, chest, and abdominal CT scans were reviewed, and the efficacy of the treatment was evaluated as “partial remission.” Notably, 3 months after diagnosis, following the completion of the third cycle of chemotherapy + immunotherapy, the patient developed generalized erythema with itching, burning skin, and multiple blisters all over the body, which dried up and caused pain at the rupture site. The patient sought treatment in the Department of Oncology of our hospital in the fourth month after diagnosis. The patient had not used any other medication during the course of the disease and denied a history of drug or food allergies. A previous history of more than 40 years of smoking was noted, with an average consumption of 20 cigarettes per day and no smoking cessation. No other adverse effects were observed.

### 2.2 Treatment and regression

**Figure 1:**
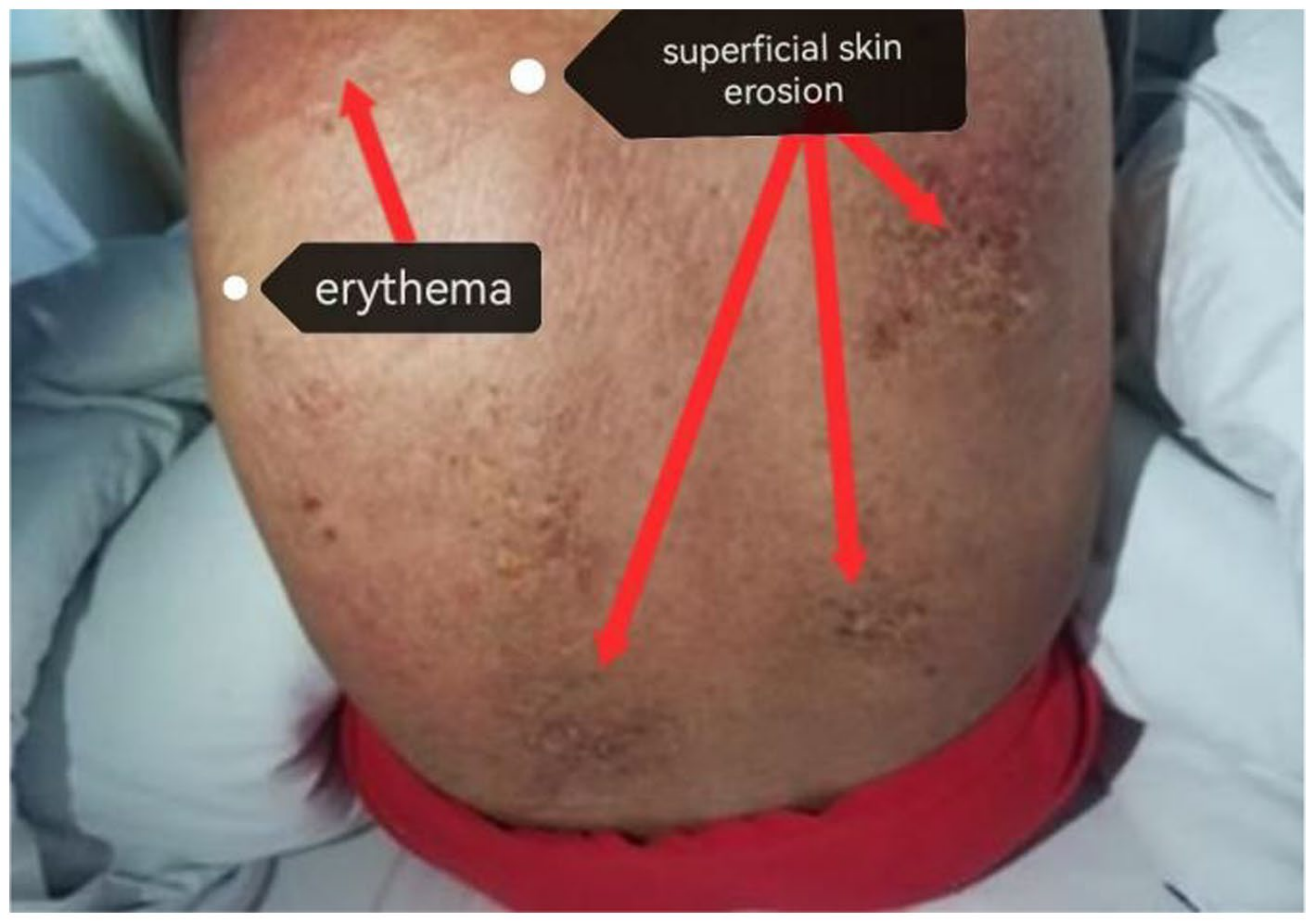
Erythema and superficial erosion of the back (first week of admission).

During the first week of admission, diffuse erythema was observed over the entire body, with superficial vesicles on the back, accompanied by tenderness and exudation. A large amount of desquamation was observed on the limbs, and Nikolsky’s sign was positive on the hips. The lips and mucous membranes were swollen, while the mucous membranes showed no abnormality, and the skin involvement area was more than 80%. Combined with traditional Chinese medicine (TCM) diagnosis and treatment and the four diagnoses, the condition was identified as “Dampness and Poison in the Skin” (“Dampness” refers to an increased vascular permeability leading to the leakage of tissue fluid, lymphocytes, proteins, and other substances into the interstitial spaces or onto the skin surface, manifesting as blisters, swelling, erosion, and exudation; “Unk” refers to infection and inflammation presenting as redness, swelling, heat, and pain; and “In the skin” refers to a concentrated outbreak at the skin level) (21). The TCM treatment principle was “clearing heat and eliminating dampness and toxins,” and the prescription was “castor seed and dampness seepage soup plus subtraction,” taken warm in the morning, noon, and night. The patient was also administered oral antihistamines, including loratadine and ebastine tablets (22) (Figure 1).

**Figure 2:**
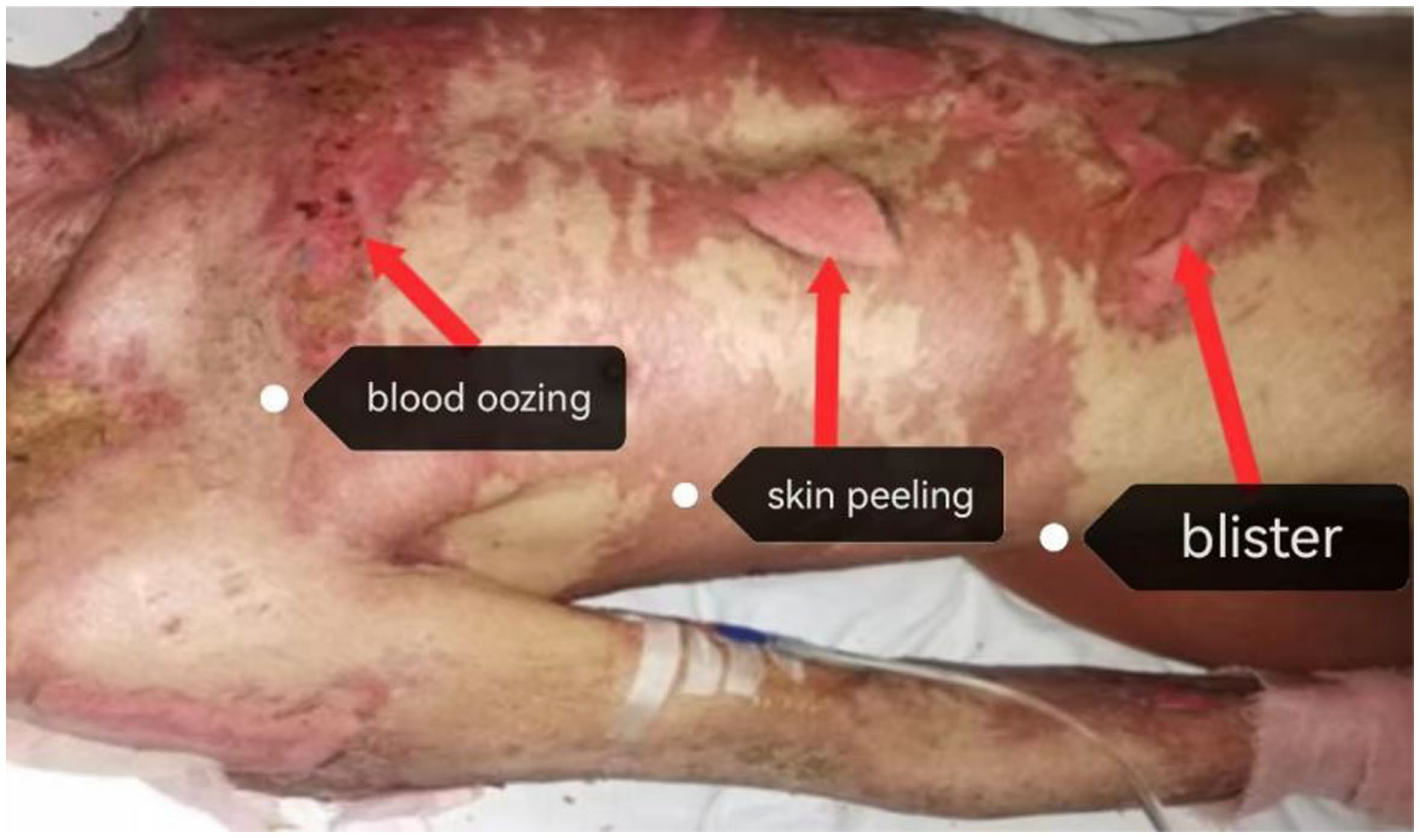
Large peripheral skin peeling with blood oozing (second week of admission).

In the second week of admission, the patient had large diffuse dark red patches on the face, trunk, and limbs, on which vesicles could be observed. Vesicles of varying sizes, blood crusts, and exudation were observed on the lips, mouth, and external genitalia, along with a large area of peripheral skin peeling off together with oozing blood. Based on traditional Chinese medicine (TCM) diagnosis, this was identified as “Fire Toxin Invading the Interior Syndrome” (“Fire Toxin” refers to a bacterial infection accompanied by the release of large amounts of bacterial toxins into the bloodstream and “Invading the Interior” refers to the progression of a localized infection into a systemic infection) (21). The prescribed TCM treatment principle was “clearing the camp and cooling the blood, opening up and detoxifying the body,” and the prescription was “detoxification and cooling the blood soup plus subtraction” (22). Simultaneously, the patient received an intravenous injection of cefozoxime sodium to prevent infection and an intravenous injection of human immunoglobulin to improve the immune system (Figure 2).

**Figure 3:**
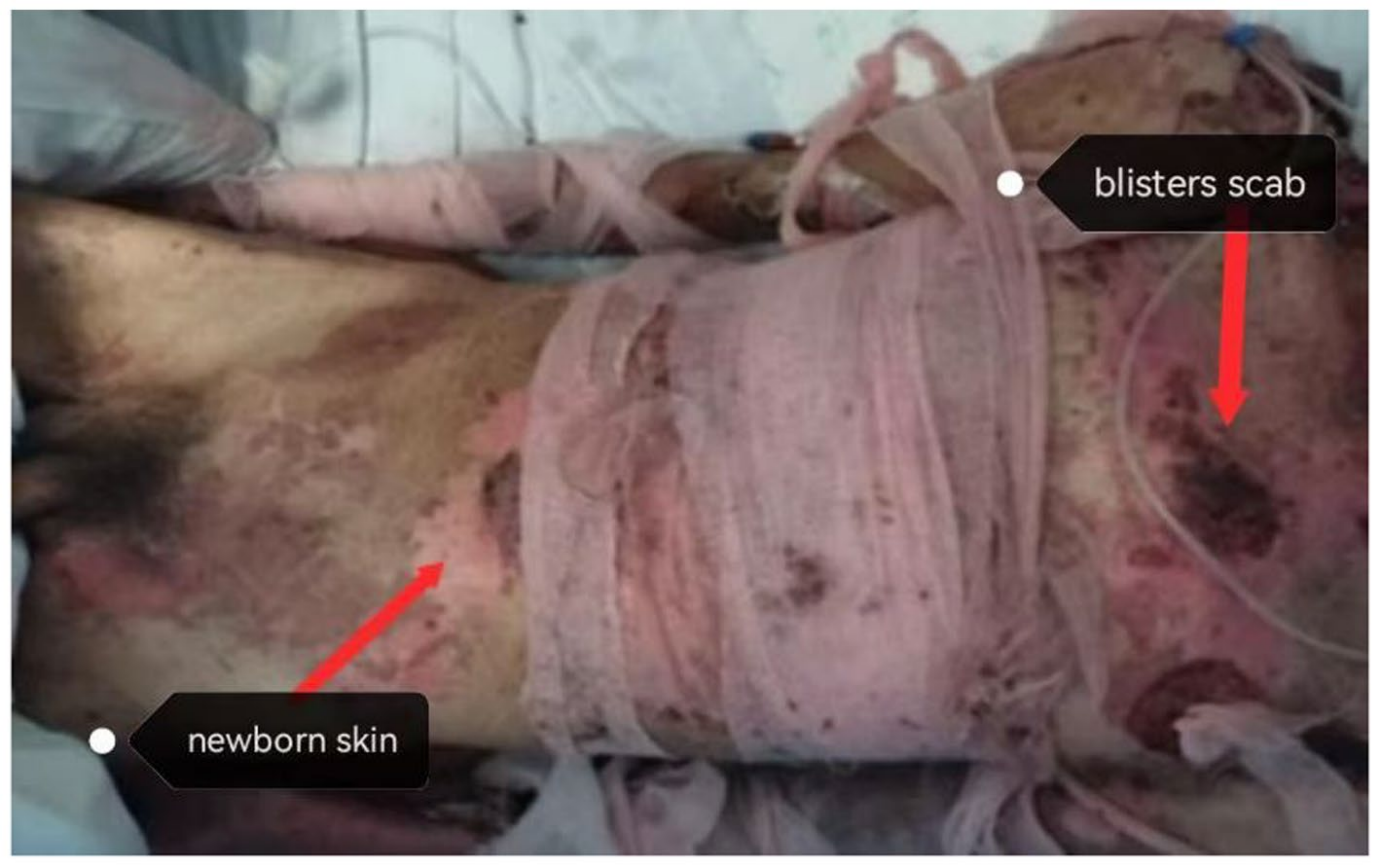
Erythema darkened significantly, blisters dried up, crusted, flaked, and new skin appeared (third week of admission).

In the third week of admission, the patient’s erythema had obviously darkened. Blisters had dried up, scabbed, and flaked, revealing new skin, and there was no obvious exudation. Based on TCM diagnosis and the four other diagnoses, the patient was identified as having “Spleen Deficiency and Yin Injury Syndrome” (“Spleen Deficiency” refers to a diminished capacity to convert food into energy and nutrients, and “Yin Injury” refers to a fluid and electrolyte imbalance accompanied by a reduction in tissue fluids and secretions) (21). The treatment principle was to “replenish qi to strengthen the spleen and benefit the stomach to nourish the yin,” and the prescribed formula was “Ginseng Ling Bai Zhu San plus reduction” (22) (Figure 3).

2.2.4 In the fourth week of admission, the majority of scabs on the head, face, trunk, and limbs had fallen off. The original vesicles on the external genitalia had completely healed, with no new lesions observed. Oral TCM treatment, intravenous albumin, and other supportive treatments were continued. After more than 1 month of hospitalization, all skin lesions had healed, the body temperature had stabilized, and the patient reported no discomfort.

## 3 Discussion

Immunosuppressants are monoclonal antibodies that activate the immune system to enhance antitumor immunity. However, immunotherapy-related adverse reactions typically affect multiple organs and systems (23). During immunotherapy, T cells within the body become fully activated. Satisfactory therapeutic effects can be achieved only when these activated T cells extensively infiltrate the tumor. Research has indicated that PD-1 inhibitors may lead to excessive T-cell activation, inducing the release of immune-related pro-inflammatory factors that contribute to the onset and progression of skin adverse reactions. This activation may be key in triggering severe TEN reactions, although the specific molecular and cellular mechanisms require further investigation (11). On the other hand, PD-1 inhibitors can increase sensitivity to radiation and chemotherapy. When PD-1 inhibitors are combined with radiotherapy or chemotherapy, enhanced monitoring of adverse reactions is required (24). Immunotherapy disrupts the previously balanced immune system, leading to a series of immune-related adverse reactions. These reactions differ fundamentally from those associated with traditional chemotherapy and targeted drugs, and some severe immune-related adverse reactions may be fatal (9). Therefore, a thorough understanding of the mechanisms underlying immune-related adverse reactions, a mastery of the characteristics of their occurrence, and close monitoring of patient responses during treatment are crucial for the nursing management of adverse reactions following immunotherapy and the development of effective nursing interventions.

### 3.1 Skin lesion management

#### 3.1.1 Nursing care of skin lesions

The dressing change plan for skin lesions is key to skin lesion management. According to the assessment of skin lesions, skin progression can be divided into an erythematous blistering stage, a skin peeling stage, and an epithelial crawling stage; therefore, the development of a scientific and detailed drug change program and the implementation of targeted nursing measures are important initiatives. Owing to the rarity of this disease, few prospective studies have analyzed the specific adjuvant efficacy of drugs for TEN, resulting in the absence of specific treatment standards. Currently, a widely accepted approach emphasizes supportive therapy and wound care (25). However, the efficacy of surgical debridement is controversial. Evidence suggests that surgical debridement combined with drug adjuvant therapy can significantly alleviate symptoms and improve the prognosis. Glucocorticoids were the earliest effective drugs for treating TEN. The early administration of adequate glucocorticoid doses can control skin lesions, reduce inflammatory exudation, shorten disease duration, and decrease mortality (26). However, long-term steroid use increases the risk of skin and mucosal infections and leads to disease recurrence. Therefore, this study used surgical debridement combined with adjunctive medicine (TCM).

Traditional Chinese medicine’s holistic theory views the human body as an integrated system where physical form and mental state (emotions and consciousness) mutually influence each other and are inseparable. Illness often manifests as a dual imbalance of “form” (physical symptoms) and “spirit” (emotional) (27). Single therapies often struggle to address both aspects simultaneously. Therefore, oral therapies primarily regulate the internal organs’ qi and blood. External therapies directly target meridians, skin regions, and acupoints to unblock local qi and blood circulation on the body surface. Music therapy directly regulates the mind and spirit, calming emotions (20). Combining multiple TCM therapies enables simultaneous intervention at different levels (physiological and psychological) and through diverse pathways (oral absorption, skin penetration, and psychological induction). This approach shortens treatment duration, enhances efficacy, and reduces the side effects and limitations of single therapies.

More than 80% of the patient’s skin was involved, making infection prevention a key priority. Therefore, the patient was transferred to a single room, and the room was disinfected with ultraviolet light twice a day for 30 min each time. The temperature and humidity of the room were suitable, ventilation was provided for 30 min a day, and the floor and surfaces of objects were wiped twice a day with a 1,000 mg/L solution of chlorine-containing disinfectant. Sterile bedclothes and sheets were changed every day to create a good environment in the hospital room. In the first week of admission, the patient had diffuse erythema all over the body, and superficial erosion was observed based on erythema on the back, accompanied by tenderness and exudation. *Angelica sinensis* and six yellow soups were chosen for external application to the lesions to clear away heat and dampness, detoxify the toxin, and alleviate pain (22). Comfrey oil was applied externally to the inguinal and axillary areas to clear away heat and detoxify the toxin (22). One week later, the patient’s erosive surface showed no enlargement, tenderness had decreased, and exudate had diminished. In the second week of admission, the patient’s skin progressed to the erythema blister stage. For blisters with a diameter of 2 cm and above, iodine povidone was used to disinfect the blisters and surrounding skin. A 1-mL sterile syringe needle was connected to a 5-mL sterile syringe, and the blisters were carefully punctured along the root of the blisters in a direction parallel to the skin. The blisters were then gently squeezed with a sterile swab to preserve the epidermis as much as possible and to minimize local injury. Small blisters with a diameter of <2 cm should be protected to promote their self-absorption, and large blisters with loose epidermis that have not been detached should not be removed from the blister skin but can be retained in the original place to act as biological dressings (28). If the epidermis is detached, necrotic, and adheres to the wound in piles, the easily removable portions can be removed directly, whereas the areas that are difficult to remove should not be forcibly removed and can be cut with sterilized scissors (29). Following the above skin care regimen maximizes preservation of the patient’s skin barrier function. During this period, the patient developed no secondary skin infections. Skin erythema noticeably faded, while vesicles and bullae dried, crusted over, and desquamated. New skin emerged with no significant exudation observed. During the fourth week of admission, the patient entered the epithelial crawling period. During this stage, recombinant human epidermal growth factor was applied to the skin lesion surfaces to accelerate the growth of granulation tissue and epithelial cell proliferation (30). In addition, the newly formed skin tissue was carefully protected to avoid dragging, pulling, tugging, and other actions. At this stage, the majority of scabs on the patient’s head, face, trunk, and limbs had fallen off. The original erosive lesions on the external genitalia have completely healed, with no new skin lesions developing.

### 3.2 Pain management

#### 3.2.1 TCM for pain relief

The beneficial stimulation created by herbal compresses on the painful areas and surrounding acupoints helps regulate the flow of qi and blood within the meridians, alleviating muscle spasms and tissue adhesions to improve pain at a functional level (31). This therapy combines the pharmacological effects of herbs with the physical warmth of heat, synergistically promoting local blood circulation and accelerating the elimination of metabolic waste, thereby achieving a multipathway intervention for pain symptoms (32). Clinical practice has shown that herbal compresses provide an effective adjunctive treatment for various acute and chronic pain conditions, including soft tissue injuries, arthritis, and postoperative pain. This therapy offers a relatively safe administration and flexible formulation option, making it a valuable component of multimodal pain management strategies (31). Skin itching and pain are often associated with “blood deficiency with wind-dryness” and “damp-heat accumulation in the skin.” The *Angelica six yellow soup*, composed of herbs that nourish yin and blood while clearing heat and drying dampness, precisely addresses these pathomechanisms (33). *Angelica sinensis* replenishes and activates blood circulation, thereby improving skin microcirculation and nutritional status. *Radix Rehmanniae Praeparata* nourishes yin and moistens dryness, alleviating itching caused by dryness. *Scutellaria baicalensis*, *Coptis chinensis*, and *Cyperus rotundus* clear heat and dry dampness, thereby reducing inflammatory responses; *Astragalus membranaceus* fortifies the exterior and strengthens the skin barrier (34). This formula embodies the TCM therapeutic principle of “nourishing blood to moisturize the skin while clearing heat and drying dampness.” *Angelica six yellow soup* exhibits significant clinical efficacy in treating patients with pruritus and skin pain (35). Peng achieved satisfactory therapeutic outcomes with high safety profiles when applying this formula to dermatological conditions such as pruritus and pain (34). Liu (33) conducted clinical observations on pruritus patients treated with *Angelica six yellow soup*, reporting marked therapeutic effects.

In the first and second weeks of admission, patients with superficial skin erosion with tenderness were treated with Chinese medicine wet compresses using *Angelica six yellow soup.* The medicinal ingredients included *Angelica sinensis*, *Scutellaria baicalensis*, *Coptis chinensis*, *Cyperus rotundus*, *Radix Rehmanniae Praeparata*, and *Astragalus membranaceu*s. This therapy was primarily used to reduce swelling and relieve pain, inhibit and prevent infections, astringe and stop itching, and promote the healing of trauma (22). The procedure (36) involved applying wet compresses with four layers of gauze. The gauze should be applied smoothly over the affected skin area, with edges extending approximately 3-4 centimeters beyond the lesion. Apply drops to the dressing every 10 minutes to maintain moisture, twice daily. One course of treatment lasted 5 days and was used for managing skin scabs and flaking. Chinese medicine wet compression therapy can make liquid pass through the skin, have a pore effect on the whole body, clear dampness and heat, dredge qi and blood, and stop itching and pain. Following treatment, the patient experienced significant relief from skin pain and itching, with scabbing and desquamation occurring as new skin formed.

#### 3.2.2 Western medicine treatment for pain relief

In the third week of hospitalization, during the patient’s skin peeling period, pain was prominent. The Numerical Rating Scale (NRS) was 9 points, and the area of epidermal loosening and peeling exceeded 56%. For effective pain relief, the patient was instructed to take oral tramadol extended-release tablets twice daily, and subcutaneous morphine was administered as needed for severe pain. Following treatment, the patient’s pain was relieved, with a NRS score of 3.

### 3.3 Management of five-element music therapy in emotions and spirits

Itching and pain often stem from insufficient liver blood, excessive liver fire, and impaired qi and blood circulation. The nature of skin itching and pain frequently plunges patients into excessive worry, which, in turn, damages the spleen. Once the spleen is impaired, it inevitably depletes qi and blood, adversely affecting the patient’s overall health (37). Selecting tranquil music in the “Yu” nourishes kidney yin and calms excessive liver yang, thereby alleviating itching. Fluid, melodious music in the “Jue” or “Zhi” modes regulates the movement of qi, promotes qi and blood circulation, and relieves pain caused by stagnation. Steady, harmonious music in the “Gong” or “Shang” strengthens spleen and stomach function, indirectly achieving qi-nourishing, blood-tonifying, and pain-relieving effects (37). In recent years, the efficacy of five-element music therapy has also been validated in treating skin diseases. Weng (20) applied this therapy to care for patients with TEN. Their emotions gradually stabilized, qi flow improved, and symptoms such as heat toxin and blood stasis were alleviated, thereby promoting recovery.

Pentatonic music therapy is a form of music therapy based on TCM fundamental theories for organ differentiation. It centers on the concept of nurturing both the body and spirit, adhering to the therapeutic principles of tailoring music to the individual, season, and specific condition. This approach uses music in five distinct pitches—“Gong, Shang, Jue, Zhi, and Yu”—to intervene in disease management (38). TCM emotional care is a psychosocial nursing model with the distinctive characteristics of Chinese medicine. It uses psychological counseling grounded in the TCM pattern differentiation theory, exhibiting positive effects in alleviating the patients’ negative emotions (39). Research (40) has indicated that TCM five-tone therapy helps regulate the human qi mechanisms and emotional states, improving anxiety and depression in patients with cancer. Patients with generalized skin eruptions, limited disease awareness, and severe anxiety were administered five-element music therapy to balance yin and yang, regulate qi and blood, and balance the dynamics of qi and qi. The doctor-in-charge of treatment prescribed music in the different keys of “Gong, Shang, Yu, Jiao, and Zheng” according to the patient’s symptoms at different times. Listening to the five elements of music while regulating the patient’s qi and blood can have a calming effect on the mind, divert attention, and relieve the discomfort caused by skin pain. Operation method: Keep the ward environment quiet, limit visits from family and friends, and ensure a chaperone is present. Assist the patient in taking a comfortable position and have them gently close their eyes. Avoid performing nursing operations during the session. Keep the volume at 30–40 decibels and guide the patient to follow the rhythm of the music using breathing relaxation and imagery training. If the patient experiences any discomfort during the treatment, the medical staff should be informed promptly. Following treatment, the patient’s anxiety levels decreased, and the discomfort caused by skin tenderness was alleviated.

### 3.4 Fluid loss management and nutritional support

Extensive skin lesions cause significant fluid loss, making the maintenance of fluid and electrolyte balance particularly critical. While providing active fluid support, it is essential to prevent secondary harm from fluid overload (41). Given the patient’s hypoalbuminemic state, human albumin infusion was administered to increase the colloid osmotic pressure, supplemented by appropriate plasma transfusion to ensure effective circulating blood volume and maintain fluid balance. This patient typically requires 10–20% more fluid replacement than patients with equivalent burn areas, with the urine output controlled at 50–80 mL/h (42).

Studies have indicated that early enteral and parenteral nutrition in patients with TEN promotes and maintains the integrity of intestinal mucosal structure and barrier function. This approach effectively corrects malnutrition, enhances immune function, reduces the incidence of related complications, lowers the mortality rates, and improves prognosis (43). The nutritional assessment tool NRS2002, introduced by the European Society for Enteral Parenteral Nutrition in 2002, is recommended as the preferred tool for nutritional risk screening of hospitalized patients in Europe. The patient had orofacial erosion, difficulty in eating, poor appetite, extreme emaciation, and a nutritional status score of 4, requiring nutritional support intervention. The medical staff established central venous access via peripheral placement and infused amino acids and fat milk to improve nutrition, to promote recovery and healing of the body, and to maintain good strength and functional status. During the second week of nutritional support, the patient exhibited a significant reduction in bleeding and exudate at the skin lesion sites, with an effective 20% decrease in the affected area. Additionally, the patient gained 1.5 kg in body weight compared to previous measurements.

## 4 Research limitations

(1) This study reports only one patient’s care, resulting in limited robustness and generalizability of findings. Future prospective or retrospective studies with larger sample sizes are needed to validate and support these results. (2) This study lacks organized biochemical data for the patient, and no long-term follow-up data were collected after the patient’s discharge following treatment completion. Future research should consider longitudinal tracking and refine multi-faceted observational indicators to provide more comprehensive scientific evidence.

## 5 Summary

With the widespread clinical application of tumor immunotherapy, immune-related adverse events (irAEs) with unclear mechanisms are becoming a significant challenge affecting the quality of life and long-term survival of patients. Against this backdrop, the integrated diagnostic and therapeutic pathways of Chinese and Western medicine exhibit unique advantages. Even when confronted with specific adverse reactions rarely reported in clinical studies, this model enables timely pathogenesis analysis and syndrome differentiation through the systematic collection of TCM diagnostic information, thereby rapidly formulating personalized treatment plans. Practice has shown that such interventions promote clinical symptom relief, control disease progression, and improve prognosis. TCM encompasses a wide range of therapeutic approaches, including not only oral decoctions but also diverse formulations, such as topical preparations and herbal injections. With accurate pattern identification, the judicious selection of non-oral routes of administration often results in rapid therapeutic effects. Therefore, clinical practice should not rigidly adhere to traditional medication patterns but should focus on exploring synergistic mechanisms among different treatment modalities. This field requires rigorously designed clinical trials to provide high-level medical evidence for the integrated application of multiple TCM therapies, thereby advancing scientific development and clinical implementation. This study presents experience in integrated Chinese and Western nursing care for toxic epidermal necrolysis, aiming to provide reference and insights for future nursing professionals managing related conditions. It aims to refine nursing protocols and contribute traditional Chinese nursing perspectives toward establishing high-quality care pathways for skin-related adverse reactions in cancer patients.

## Data Availability

The original contributions presented in the study are included in the article/supplementary material; further inquiries can be directed to the corresponding author.
